# The mediating effect of body appreciation between muscle dysmorphia and bulimia nervosa among Lebanese adolescents

**DOI:** 10.1186/s12888-023-04772-2

**Published:** 2023-04-20

**Authors:** Deema Rahme, Mariam Dabbous, Diana Malaeb, Souheil Hallit, Sahar Obeid

**Affiliations:** 1 Pharmacy Practice Department, Faculty of Pharmacy, Beirut Arab University, Beirut, Lebanon; 2 School of Pharmacy, Lebanese International University, Beirut, Lebanon; 3 College of Pharmacy, Gulf Medical University, Ajman, United Arab Emirates; 4 School of Medicine and Medical Sciences, Holy Spirit University of Kaslik, P.O. Box 446, Jounieh, Lebanon; 5 Research Department, Psychiatric Hospital of the Cross, Jal Eddib, Lebanon; 6 Applied Science Research Center, Applied Science Private University, Amman, 11931 Jordan; 7 Social and Education Sciences Department, School of Arts and Sciences, Lebanese American University, Jbeil, Lebanon

**Keywords:** Bulimia Nervosa, Body dysmorphic disorder, Body appreciation, Adolescents, Lebanon

## Abstract

**Background:**

Muscle dysmorphia (MD) is a common psychological disorder present in Lebanese adolescents. Several disorders can be present concomitantly with MD such as bulimia nervosa and exercise addiction. This study aims to explore the mediating effect of body appreciation between Muscle dysmorphia and bulimia nervosa, and to validate the exercise addiction inventory scale in Lebanese adolescents.

**Methods:**

This was a cross-sectional designed study, conducted between August-September 2022, and enrolling 403 adolescents currently residing in Lebanon (15 to 18 years old), from all Lebanese governorates (Beirut, Mount Lebanon, North, South, and Bekaa). Our sample was chosen using the snowball technique, a soft copy of the questionnaire was created using google forms software, and an online approach was conceived to proceed with the data collection.

**Results:**

The mean age of the participants was 16.63 ± 1.46 years with 57.3% females. The factor analysis for the Exercise Addiction Inventory suggested one factor, which explained 62.58% of the common variance. The confirmatory factor analysis revealed an adequate fit to the model with satisfactory Maximum Likelihood Chi-Square/Degrees of Freedom (χ^2^/df), SRMR, CFI, TLI, and AVE. The mediation analysis showed that the direct relation between bulimia and MD was not significant after eliminating the effect of body appreciation (r = .12, p < .001). However, body appreciation was significantly associated with bulimia nervosa (r=-1.06, p < .001) and MD (r=-.28, p < .001).

**Conclusion:**

Body appreciation was found to be a mediator between muscle dysmorphia and bulimia nervosa and thus it is the main factor leading to both disorders. Therefore, body appreciation should be addressed in the evaluation and management of Muscle Dysmorphia.

## Introduction

Muscle Dysmorphia (MD) is a form of Body Dysmorphic Disorder (BDD). This disorder is characterized by a pathological belief that one’s body is looking too small and insufficiently muscular despite having a normal body shape. However, in females, it refers to both being insufficiently muscular and lean [1]. MD occurs mainly during adolescence as a consequence of social pressure regarding perfect body image [2]. The prevalence of MD among college students was approximately 6%. However, the disorder occurs in approximately 15 to 45% of weightlifters [3].

The causes of MD are uncertain; yet several factors such as biology, self-concept, social pressure, and the impact of media which is shaping the concept of beauty among teenagers and adults and negatively influencing one’s perception of his/her own body which can lead to unhealthy behaviors and various psychological disorders [4].

Those affected by MD usually adopt a restrictive lifestyle characterized by excessive physical exercise and a rigid diet [5]. It is very common in muscle dysmorphia to follow a meticulous diet, which usually contains high protein and low fat, and to exercise strenuously to the extent that may damage their joints or muscles. Moreover, these individuals may abuse a large variety of drugs such as thyroid hormone, selective estrogen receptor modulators, androgenic steroids, and human growth hormone, aiming to lose fat and build up muscle. However, the body-modifying behaviors to achieve the ideal muscularity in MD patients are quite similar for males and females. While muscularity is primarily achieved through exercise, the level of thinness is often regulated by caloric intake. Studies have shown that the drive for muscularity and the drive for thinness are concurrently present in both males and females and are associated with Eating Disorders (ED) [6, 7]. Binge eating occurs in a wide range of EDs. It is a preliminary diagnostic feature of Bulimia Nervosa [8]. Knowing that the perfect muscular appearance is usually associated with periodic recurrent eating ‘bulking’ to build muscle mass, MD might lead to high levels of overeating, loss of control, and thus binge eating [9]. Hence, MD psychopathology is positively associated with Bulimia Nervosa in both men and women [10].

Despite the positive correlation between MD and EDs, including bulimia nervosa, reported in previous studies [11], the similarities between these disorders are worth further investigation. The clinical features of both disorders are overlapping. Foremost, they are both characterized by low levels of body appreciation and dissatisfaction about the body size and shape. In this context, the body is considered not muscular enough for males, not lean for females, and never skinny enough in both disorders [10]. Nevertheless, cognitive distortion about physical appearance and low self-esteem are considered etiological factors for both MD and bulimia nervosa symptoms in both genders [11, 12]. In this context, several studies have found that body appreciation is consistently associated with lower levels of eating disorder symptoms, body image concerns, mental health problems, and higher levels of self-esteem, well-being, and self-compassion [13, 14]. Hence, body appreciation is considered a crucial intervention to be targeted in mental health promotion initiatives and eating disorder prevention programs.

Many psychological disorders are associated with the idea of a socially idealized body. Body dissatisfaction is fairly prevalent in our society where unrealistic and unattainable body ideals are prevalent. Women who are comparing themselves to social ideals of the slim body experience dissatisfaction with their bodies, whereas men experience feelings of dissatisfaction when they perceive that their bodies do not measure up to social ideals of having a muscular body, as well as the notion that this ideal shape is unattainable by most men and therefore highly valued [15, 16]. These issues with low body appreciation can also lead to muscle dysmorphia.

Exercise addiction (EA) is among the disorders that are associated with MD as reported in previous studies which revealed that people with MD showed the highest levels of EA, exercising longer and more frequently in comparison with people without MD [17, 18]. Likewise, when MD increased, there was a greater risk of EA, which favored body dissatisfaction and the exacerbation of MD [19]. Exercise addiction is not frequently diagnosed by healthcare providers because exercise is well-known to be a healthy habit and is difficult to recognize it as an unhealthy obsession. Moreover, the tools that are available to diagnose EA are time-consuming when administered to patients, their scoring sometimes is complicated, and their interpretation is not always clear. However, The Exercise Addiction Inventory (EAI) is a short, valid, and reliable instrument used to assess the risk for exercise addiction, and has been implemented as a screening tool for EA in previous studies [20, 21]. The EAI consists of six items scored on a 5-point Likert scale which is based on the components model of addiction as the theoretical base for this scale [22]. Because EAI is a quick and feasible tool for diagnosing EA, it has been translated and validated in several different languages (e.g., Chinese [23], Danish [24], French [25], Italian [26], and Spanish [27]) for a reliable implementation. Nevertheless, EAI has a limitation which is that the rating on its Likert scale is not incremental but varies from “strongly disagree” to “strongly agree,” with a midpoint response of “neutral,” which gives a score of 3/5 which can arbitrarily increase the total score of the EAI. Therefore, the Revised Exercise Addiction Inventory (EAI-R) was developed which includes the six items rated on a 6-point Likert scale (1 = strongly disagree, 2 = disagree, 3 = slightly disagree, 4 = slightly agree, 5 = agree, and 6 = strongly agree) to eliminate the midpoint uncertainty from the original scale [28].

Eating disorders are considered among the most severe and prevalent mental disorders in adolescents and young adults in both Western and non-Western societies, including Lebanon. As reported in a previous study, bulimia nervosa is the most prevalent ED (46.1%) followed by anorexia nervosa (39.4%) and binge eating (14.4%) [29]. Several factors are associated with the prevalence of ED in the Lebanese population. Higher Body Mass Index (BMI), depression, anxiety, greater body dissatisfaction, and pressure from media to lose weight are the most commonly associated with higher ED [30]. Social media has a great influence on the Lebanese population and it often perpetuates the perfect thin body image which has been linked to body dissatisfaction, especially among young females. Moreover, social pressure plays an important role in lower body appreciation which can lead to eating disorders.

However, studies investigating the factors associated with MD and Bulimia Nervosa among adolescents in Lebanon are lacking. A study exploring the factors associated with body dissatisfaction among the Lebanese population showed that ED, binge eating, was significantly associated with higher body dissatisfaction [31. However, the factors associated with MD in adolescents have not been addressed in any previous study in Lebanon. Therefore, it is essential to investigate the factors and the underlying disorders that are associated with MD in order to raise awareness of this disorder which may be undetectable by healthcare professionals. Moreover, a better understanding of MD will aid in the implementation of effective screening and management programs later on to protect the health and well-being of the Lebanese youth population from the harmful consequences of this disorder. Hence, this study aims to investigate the factor associated with MD and the mediation effect of body appreciation between MD and Bulimia Nervosa among Lebanese adolescents. As a secondary objective, the study also validates the Exercise Addiction Inventory questionnaire in the Lebanese population.

## Methods

### Study Design

This was a cross-sectional designed study, conducted between August-September 2022, and enrolling 403 adolescents currently residing in Lebanon (15 to 18 years old), from all Lebanese governorates (Beirut, Mount Lebanon, North, South, and Bekaa). The snowball method was used to choose our sample, Google Forms was used to make a soft copy of the questionnaire, and an online approach was developed to continue with the data collecting. Prior to their participation, participants received online instructions on how to complete the questionnaire as well as the primary aims and objectives of the study. Later, initial participants were asked to recruit other participants they know, preferably as diverse as possible with regard to place of habitat within the Lebanese governorates and within the same age interval required to participate in the study. There were no credits received for participation.

### Participants

A total of 403 adolescents participated in this study; their mean age was 16.63 ± 1.46 years, with 57.3% females; 17 (4.2%) of the adolescents showed exercise addiction. Other characteristics are summarized in Table 1. The sample was later divided into two subsamples using the SPSS select cases option, to ensure adequate sample sizes for both exploratory and confirmatory factor analysis; resulting in one split-half for EFA (women *n* = 109, men *n* = 89) and a second split-half for CFA (women *n* = 122, men *n* = 83). There were no significant differences between the two subsamples in terms of mean age, *t*(401) = 0.08, *p* = .935, and BMI, *t*(401) = 0.07, *p* = .944, as well as sex, χ^2^[1] = 0.82, *p* = .365 and education χ^2^[1] = 0.50, *p* = .480.

**Table 1:** Sociodemographic and other characteristics of the participants (N = 403)

Variable	N (%)
Sex	
Male	172 (42.7%)
Female	231 (57.3%)
Education	
Complementary	79 (19.6%)
Secondary	324 (80.4%)
	**Mean ± SD**
**Age (in years)**	16.63 ± 1.46
**Household crowding index (persons/room)**	1.15 ± 0.56
**Body Mass Index (kg/m** ^**2)**^	22.14 ± 4.14
**EAT bulimia**	3.54 ± 4.60
**Muscle dysmorphic disorder**	12.97 ± 9.14
**Exercise addiction**	15.04 ± 5.09
**Body appreciation**	36.77 ± 10.31

### Ethics approval and consent to participate

The Ethics and Research Committee of the Lebanese International University approved this study protocol. Informed consent was obtained from the parents of all children participants. All methods were performed in accordance with the relevant guidelines and regulations.

### Minimal sample size calculation

A minimal sample of 125 was deemed necessary using the formula suggested by Fritz and MacKinnon to estimate the sample size: $n = \frac{L}{f^{2}}+k+1$, where f=0.26 for small-to-medium effect size, L=7.85 for an α error of 5% and power β = 80%, and k=8 variables to be entered in the model.

### Questionnaire

A questionnaire in Arabic was sent as a Google form link via social media networks. It needed 7–10 min to be filled. It contained sociodemographic information about the participants (age, gender, governorate, current self-report weight and height). The Body Mass Index (BMI) was consequently calculated as per the World Health Organization [32]. The household crowding index, reflecting the socioeconomic status of the family, is the ratio of the number of persons living in the house over the number of rooms in it (excluding the kitchen and the bathrooms) [33]. The second part included the scales used:

*Eating Attitudes Test (EAT-26)* is used to assess Bulimia, our independent variable. The EAT, validated in Arabic [34, 35], was used to identify the presence of “eating disorder risk” based on attitudes, feelings and behaviors related to eating. The scale has three subscales: Dieting, Bulimia and Food Preoccupation, and Oral Control [36]. It includes seven items derived from the EAT-26 (Items 2,6,7, 15, 16, 17 & 22) each with six response options, ranging from infrequently/almost never/ never (0) to always [3]. Higher scores indicates inappropriate eating behaviors. All subscales can be added to give a total score, or each subscale can be used independently (McDonald’s ω in this study = 0.91).

*Muscle Dysmorphic Disorder Inventory (MDDI)* is used to assess the MDD, our dependent variable. *The MDDI* is a 13-item questionnaire that contains three subscales directly related to MD: “drive for size” used to measure the desire to increase muscle mass, “appearance intolerance” used to measure the desire to lose fat mass, and “functional impairment” used to measure avoidance of social situations [37]. Respondents rate statements on a 1 (never) to 5 (always) scale. The three subscales’ scores are: Drive for Size (DFS, 5 items, range 5–25), Appearance Intolerance (AI, 4 items, range 4–20), and Functional Impairment (FI, 4 items, range 4–20) [37]. These three scores yield to a total possible MDDI score ranging from 13 to 65 and the cut-off score is 39 where participants having a score greater than this are classified as subjects with MD symptoms [38] (McDonald’s ω in this study = 0.85). The Arabic validated version was used in this study [39].

*Body Appreciation* Scale is used to assess the mediating variable (body appreciation). This 10-item instrument assesses acceptance of one’s body, respect and care for one’s body, and protection of one’s body from unrealistic beauty standards [40. Items are rated on a 5-point scale (*never* to *always*). Higher scores on this scale reflect greater body appreciation (McDonald’s ω in this study = 0.97).

*Exercise Addiction Inventory – Youth* (EAI-Y) was developed on the basis of the six components of behavioral addictions [41]. The items address: salience (exercise is very important), conflicts (large exercise amounts lead to conflicts with parents), mood modification (exercise is used to change emotions), tolerance (increasing exercise over time), withdrawal symptoms (abstinence symptoms occur when exercise is missed), conflict (compromising of relationships, education, job, etc.), and relapse (returning to excessive exercise following a period of abstinence). The items are responded on a 5-point Likert scale ranging from strongly disagree [1] to strongly agree [5]. Higher scores indicate higher exercise addiction. Values of 24 and above would reflect exercise addiction [42] (McDonald’s ω in this study = 0.88).

### Translation Procedure

The forward and backward translation method was applied to different scales. The English version was translated to Arabic by a Lebanese translator who was completely unrelated to the study. Afterwards, a Lebanese psychologist with a full working proficiency in English, translated the Arabic version back to English. The initial English version and the second English version were compared to detect and later eliminate any inconsistencies. A pilot study was done on 20 participants to make sure that all questions were understood; no changes were done afterwards.

### Statistical analysis

**Exploratory factor analysis.** To explore the factor structure of EAI-Y, we computed a principal-axis EFA with the first split-half subsample using the SPSS software v25. We verified all requirements related to item-communality [43], average item correlations, and item-total correlations. The Kaiser-Meyer-Olkin (KMO) measure of sampling adequacy (which should ideally be ≥ 0.80) and Bartlett’s test of sphericity (which should be significant) ensured the adequacy of our sample. Item retention was based on the recommendation that items with “fair” loadings and above (i.e., ≥ 0.4) and with low inter-item correlations (suggestive of low item redundancy) as indicated by the anti-image correlation matrix should be retained. A promax rotation was applied since items were highly correlated.

We used data from subsample 2 to conduct a CFA using the SPSS AMOS v.26 software. A previous study suggested that the minimum sample size to conduct a confirmatory factor analysis ranges from 3 to 20 times the number of the scale’s variables. Therefore, we assumed a minimum sample of 120 participants needed to have enough statistical power based on a ratio of 20 participants per one item of the scale, which was exceeded in this subsample. Our intention was to test the original model of EAI scores (i.e., a unidimensional model; [42]. Parameter estimates were obtained using the maximum likelihood method and fit indices. Additionally, evidence of convergent validity was assessed using the average variance extracted (AVE), with values of ≥ 0.50 considered adequate.

Composite reliability in both subsamples was assessed using McDonald’s (1970) ω, with values greater than 0.70 reflecting adequate composite reliability [43]. McDonald’s ω was selected as a measure of composite reliability because of known problems with the use of Cronbach’s α [44]. To assess convergent and criterion-related validity, we examined bivariate correlations between EAI scores and those on the additional measures included in the survey (eating attitudes, MDD and BAS) using the total sample. Based on Cohen’s recommendations [45] values ≤ 0.10 were considered weak, ~ 0.30 were considered moderate, and ~ 0.50 were considered strong correlations.

SPSS v25 was used for the remaining statistical analysis. MDD scores were considered normally distributed since the skewness and kurtosis varied between − 1 and + 1 [46]. Student t test was used to compare two means. Pearson test was used to correlate two continuous variables. The PROCESS Macro v.3.4 model 4 was used to conduct the mediation analysis [47]. Four pathways were calculated; Pathway A from the independent variable to the mediator, Pathway B from the mediator to the dependent variable and Pathways c and c’ reflecting the total and the direct effects of the independent variable on the dependent one respectively. The mediation analysis was considered significant if the Bootstrapped confidence interval of the indirect effect did not pass by zero [47]. Significance was set at p < .05.

## Results

### Validation of exercise addiction inventory by factor analysis (subsample 1)

In sample 1, the Bartlett’s test of sphericity *p* < .001, and KMO (0.852) indicated that the EAI items had adequate common variance for factor analysis. The results of the EFI revealed one factor, which explained 53.32% of the common variance.

### Factor structure congruence and composite reliability

The factor loadings reported in Table 1 suggest an appropriate factor structure among sample 1. McDonald’s ω was adequate in the total subsample (ω = 0.87).

### Confirmatory factor analysis (subsample 2)

CFA indicated that fit of the one-factor model of EAI scores was poor: χ^2^/df = 118.21/9 = 13.13, SRMR = 0.078, CFI = 0.840, and TLI = 0.733. After applying two correlations between residuals of items 1–3 and 3–4, the fit indices improved as follows: χ^2^/df = 25.82/7 = 3.69, SRMR = 0.038, CFI = 0.972, and TLI = 0.941. The standardised estimates of factor loadings were all adequate (see Table 2). The convergent validity for this model was adequate, as AVE = 0.53.

**Table 2:** Items of the Exercise Addiction Scale in English and Factor Loadings Derived from the Exploratory Factor Analyses (EFA) in the First Split-Half Subsample, and Standardized Estimates of Factor Loadings from the Confirmatory Factor Analysis (CFA) in the Second Split-Half Subsample

Item	EFA	CFA
1	0.74	0.61
2	0.63	0.62
3	0.77	0.60
4	0.85	0.72
5	0.83	0.91
6	0.83	0.85

### Composite reliability

Composite reliability of scores was adequate in sample 2 (ω = 0.87).

### Divergent validity

EAI scores were significantly and moderately associated with higher MDD scores (Table 3).

### Bivariate analysis

The results of the bivariate analysis are summarized in Tables 3 and 4. Higher bulimia and exercise addiction were significantly associated with more MDD, whereas higher body appreciation was significantly associated with less MDD.

**Table 3:** Bivariate analysis of categorical factors associated with MDD

	MDD (Mean ± SD)	T	Df	*p*
Gender		0.226	401	0.821
Male	13.09 ± 9.53			
Female	12.88 ± 8.85			
Education		1.134	401	0.257
Complementary	14.01 ± 10.06			
Secondary	12.71 ± 8.90			

**Table 4:** Correlation of continuous variables with MDD

	1	2	3	4	5	6	7
1. MDD	1						
2. Age	0.02	1					
3. Body Mass Index	0.03	0.20***	1				
4. Household crowding index	− 0.01	0.04	− 0.08	1			
5. Bulimia	0.20***	0.07	0.19***	− 0.02	1		
6. Body appreciation	− 0.35***	− 0.04	− 0.19***	0.01	− 0.47***	1	
7. Exercise addiction	0.27***	0.08	0.03	− 0.03	− 0.02	− 0.01	1

*p < .05; **p < .01; ***p < .001; MDD = Muscle Dysmorphic Disorder

### Mediation analysis

**Fig. 1:**
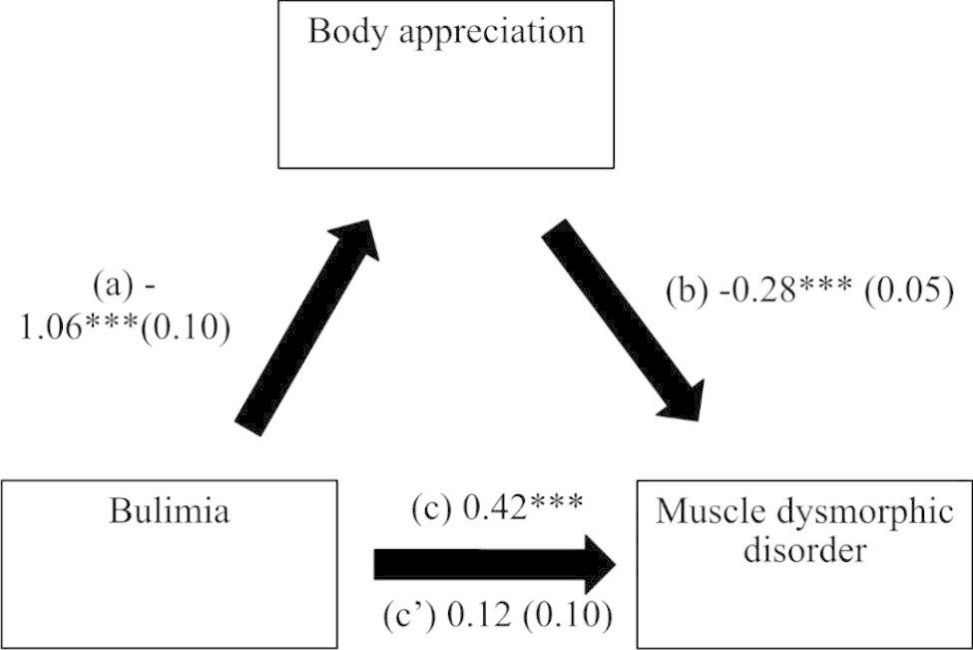
(a) Relation between bulimia and body appreciation (R^2^ = 22.38%); (b) Relation between body appreciation and muscle dysmorphic disorder (R^2^ = 19.66%); (c) Total effect of the relation between bulimia and muscle dysmorphic disorder (R^2^ = 11.89%); (c’) Direct effect of the relation bulimia and muscle dysmorphic disorder. Numbers are displayed as regression coefficients (standard error). ***p < .001

Body appreciation mediated the association between bulimia and MDD (indirect effect: Beta = 0.30; BootSE = 0.06; BootCI [0.19; 0.42]. Higher bulimia was significantly associated with lower body appreciation, whereas higher body appreciation was significantly associated with lower muscle dysmorphic disorder (Fig. 1). Finally, bulimia was not directly associated with muscle dysmorphic disorder.

## Discussion

The study results showed good psychometric properties of the one-factor model of the Exercise Addiction Inventory for youth and suggested a full mediating effect of body appreciation between bulimia and MDD; higher bulimia was associated with lower body appreciation, whereas higher body appreciation was associated with lower muscle dysmorphic disorder. Finally, bulimia was not directly associated with muscle dysmorphic disorder.

### EA Validation

The Arabic version of the EAI-Y indicated a single factor structure similar to the original scale [41] which indicates a strong factorial validity. The indices obtained showed that this Arabic version has an adequate model ft according to the CFA value. Moreover, the Cronbach’s α of the EAI-Y total scale was good (0.88), which indicates that the scale has a high internal consistency. Lower results were found in the original scale with Cronbach’s α 0.7 [41]. In the latter study, when participants were divided into the three sub-groups, the α was 0.6 in the adolescent sport school students, 0.7 in the fitness center attendees, and 0.9 in the patients with eating disorders. In the Dutch validation study, Cronbach’s alpha was 0.66 whereas, in the study by Terry et al., it was 0.84 [24, 48]. Thus, the Arabic version of the EAI-Y scale can be considered a reliable tool for screening exercise addiction in Lebanese adolescents. However, future studies should target the assessment of more psychometric properties of this scale.

### Body appreciation and BN

As expected, low body appreciation was significantly associated with Bulimia Nervosa. This is in line with longitudinal studies that demonstrated that low body appreciation is the most reliable predictor of ED [49]. Moreover, several studies also confirmed the strong correlation between body dissatisfaction and bulimia manifested by binge eating [31, 50]. This could be explained by the fact that individuals with low body appreciation and negative thoughts about their physical image are more likely to be engaged in unhealthy behaviors, such as diet, abusing weight loss medications, fasting, vomiting, and using laxatives in an attempt to reach the perceived ideal body.

### Body appreciation and MD

This study showed a significant negative correlation between body appreciation and MDD, supporting the findings from previous studies [51, 52]. A possible explanation for these findings is that individuals with lower body appreciation usually think that they are not physically fit or muscular enough and hence these unrealistic thoughts can precipitate MD as they get concerned with their perceived lack of muscularity, despite typically having above-average muscularity.

### Mediation effect

Despite the previously mentioned findings, recent research has demonstrated that indviduals desiring a perfect body image often demonstrate eating behavior disorders and low body appreciation issues [52–54]. Therefore, body dissatisfaction is not specific to MD but is a diagnostic feature for both MD and ED. To confirm this interesting finding, the mediating effect of body appreciation on MD and bulimia nervosa was statistically investigated and accordingly, the direct relation between bulimia and MD became non-significant which implies that body appreciation is the leading factor to both disorders and hence improving body appreciation can be helpful in the management of MD and Bulimia Nervosa.

### EA and MD

It is worth mentioning that despite the positive association between EA and MD in the bivariate analysis, EA was a confounding factor in our study and the mediation analysis was adjusted over it. However, this finding is consistent with previous studies [17–19]. The result can be explained by the fact that individuals with muscle dysmorphia have a delusional perception that their body is not muscular enough and is too small, despite often being more muscular than others. Many factors can contribute to this misperception such as a family history of body dysmorphic disorder or obsessive-compulsive disorder, childhood bullying, neglect or abuse, social pressure and comparison with public figures representing ideal models, the physical and hormonal changes that occur during puberty, and having another mental health condition like anxiety or depression [17–19].

This unrealistic belief about their body drives adolescents to engage in extreme exercise to pursue the muscular ideal appearance they seek. However, exercise addiction can interfere with important life activities and it may involve exercising at inappropriate times and places despite injuries, medical issues, or illness. Hence this can lead to serious health complications, mental health struggles, poor quality of life, and relationship issues. Unfortunately, excessive exercise may go unnoticed among individuals with MD since many individuals with muscle dysmorphia still appear healthy and within a normal (or above normal) weight range. Unlike EA in the case of ED where it can be easily detected as an unhealthy and harmful behavior. Hence, it is important also to address EA in cases of MD to prevent its negative impact on health and well-being.

### Clinical implications

Muscle Dysmorphia is a common disorder among adolescents and required a comprehensive assessment to detect the possible causes and underlying disorders. Several disorders should be addressed in the management of MD such as exercise addiction, Bulimia Nervosa, and body appreciation. Body appreciation is the mediator in the association between MD and bulimia nervosa, accordingly raising awareness to adjust the perception of body image and to highlight the drastic consequences of the disorders related to low body appreciation are warranted.

### Limitations

Some limitations are present in this study. First, it was based on self-reported questionnaires to assess the desired disorders (Bulimia, MD, EA) and may lead to social desirability bias. Nevertheless, not all the scales used in the study are validated in Lebanon. Second, the study is cross-sectional and hence it is not possible to draw causality relationships from the associations reported. A selection bias is present because of the inability to know the refusal rate and because of the snowball technique followed during the data collection, which does not support the generalizability of the findings. Information bias is also plausible since participants might not give honest answers to the questions. Finally, it is important to mention the possibility of residual confounding bias because other variables that might affect MDD were not considered in this study (such as time spent on social media, time spent at the gym, etc.).

## Conclusion

The positive association between muscle dysmorphia and bulimia nervosa was mediated by body appreciation which is the main factor leading to both disorders. Therefore, body appreciation should be addressed in the evaluation and management of Muscle Dysmorphia.

Moreover, public awareness should be raised about this condition in addition to preventive and remedial educational programs targeting youth and adolescents, who are at higher risk.

Such programs can be also launched on various social media aiming to correct the standards of ideal body and enhance body appreciation to prevent disorders associated with body dissatisfaction and delusional conceptualization of body image such as ED and MD.

## Data Availability

All data generated or analyzed during this study are not publicly available due the restrictions by the ethics committee. The dataset supporting the conclusions is available upon a reasonable request to the corresponding author (S.H.).
